# Atypical audio-visual speech perception and McGurk effects in children with specific language impairment

**DOI:** 10.3389/fpsyg.2014.00422

**Published:** 2014-05-20

**Authors:** Jacqueline Leybaert, Lucie Macchi, Aurélie Huyse, François Champoux, Clémence Bayard, Cécile Colin, Frédéric Berthommier

**Affiliations:** ^1^Center for Research in Cognition and Neurosciences, Université Libre de BruxellesBrussels, Belgium; ^2^Ureca, Université de Lille 3Lille, France; ^3^IPSY, Université Catholique de LouvainLouvain-la-Neuve, Belgium; ^4^École d'orthophonie et d'audiologie, Université de MontréalMontréal, QC, Canada; ^5^GIPSA-Lab, Université de GrenobleGrenoble, France

**Keywords:** multisensory speech perception, specific language impairment, McGurk effects, audio-visual speech integration, masking release

## Abstract

Audiovisual speech perception of children with specific language impairment (SLI) and children with typical language development (TLD) was compared in two experiments using /aCa/ syllables presented in the context of a masking release paradigm. Children had to repeat syllables presented in auditory alone, visual alone (speechreading), audiovisual congruent and incongruent (McGurk) conditions. Stimuli were masked by either stationary (ST) or amplitude modulated (AM) noise. Although children with SLI were less accurate in auditory and audiovisual speech perception, they showed similar auditory masking release effect than children with TLD. Children with SLI also had less correct responses in speechreading than children with TLD, indicating impairment in phonemic processing of visual speech information. In response to McGurk stimuli, children with TLD showed more fusions in AM noise than in ST noise, a consequence of the auditory masking release effect and of the influence of visual information. Children with SLI did not show this effect systematically, suggesting they were less influenced by visual speech. However, when the visual cues were easily identified, the profile of responses to McGurk stimuli was similar in both groups, suggesting that children with SLI do not suffer from an impairment of audiovisual integration. An analysis of percent of information transmitted revealed a deficit in the children with SLI, particularly for the place of articulation feature. Taken together, the data support the hypothesis of an intact peripheral processing of auditory speech information, coupled with a supra modal deficit of phonemic categorization in children with SLI. Clinical implications are discussed.

## Introduction

Children with specific language impairment (SLI) experience difficulties in understanding and producing spoken language, despite normal intelligence, normal hearing, and normal opportunities to learn language. Although linguistic deficits fundamentally characterize SLI (Bishop and Snowling, 2004), theories diverge on the causes of SLI, from grammatical deficit to general or specific limitations in processing capacities (Leonard, 1998, 2004). At the behavioral level, children with SLI are characterized by deficiencies in phonology (Bortolini and Leonard, 2000; Maillart and Parisse, 2006), morphosyntax (Leonard, 1998, 2009) and phonological short-term memory, especially in non-word repetition (Archibald and Gathercole, 2007).

The role of auditory perceptual deficits in explaining the etiology of SLI has been strongly debated. There is much controversy about whether general auditory processing deficits are important in the genesis of specific language disorders (Tallal and Piercy, 1973; Tallal, 1980) or whether the deficit is specific to speech sounds (Mody et al., 1997). Recent work suggest that there are individual differences among children with SLI regarding auditory deficits (Rosen, 2003), and that the deviants may be linked to maturity of auditory processing (Bishop and McArthur, 2004; McArthur and Bishop, 2005). A robust finding in the literature is that even if children with SLI show either no or only subtle speech perception deficits in optimal listening conditions (i.e., in quiet), they exhibit a stronger impairment than children with typical language development (TLD) in speech-in-noise perception. A speech-in-noise deficit in children with SLI has been demonstrated in English (Brady et al., 1983; Robertson et al., 2009; Ferguson et al., 2011) as well as in French (Ziegler et al., 2005, 2009).

Several hypotheses have been advanced to explain the speech-in-noise deficit (Nittrouer et al., 2011). According to a first hypothesis, children with SLI would have an auditory deficit in recovering phonetic structures because of poor sensitivity to formant transitions (Tallal, 1980; Tallal et al., 1993). This idea has been contradicted by several researchers (Sussman, 1993; Bishop et al., 1999; Nittrouer et al., 2011). A second hypothesis is that children with SLI experience more masking of these speech-relevant acoustic properties than children with TLD. According to Ziegler et al. (2009), children with language problems lack “speech robustness,” meaning that they do not have phonological representations as stable as children with TLD. Enhanced masking for speech in children with language problems could be due to those weak representations (Brady et al., 1983; Studdert-Kennedy and Mody, 1995; Johnson et al., 2009; Rosen et al., 2009; Ziegler et al., 2009). The acoustic properties needed for recovering phonetic structure could simply be masked, explaining why phonological representations are so weakly established in the first place (Wright et al., 1997). A third hypothesis is that children with SLI have more difficulties than listeners with TLD at creating well-defined and robust categories in speech as in non-speech. A phonetic category refers to the way various components of the speech signal are combined to form a linguistically meaningful percept. Creation of phonetic categories is related to phonological coding: the language users need to create well-defined categories from sensory information in the signal (Nittrouer et al., 2011).

The difficulties of children with SLI in perceiving speech sounds have been mainly studied in the auditory modality. It appeared that the reception of voicing, place, and manner is impaired in children with SLI compared to age-matched and language-matched children with TLD (Ziegler et al., 2005; but see Collet et al., 2012, for a training of voicing perception in children with SLI).

In face-to-face communication, speech perception is a multimodal process involving both auditory and visual modalities (Sumby and Pollack, 1954; Grant and Seitz, 2000). In noisy contexts, speech detection and comprehension are better in audio-visual conditions (AV), where audition is accompanied by speechreading, than in auditory-only conditions (AO), where only the auditory stimulus is present. During speech perception, auditory and visual cues are merged into a unified percept, a mechanism known as audio-visual (AV) integration. The enhancement afforded by the visual cues in speech-in-noise is largely due to the fact that vision conveys place of articulation, while audition primarily conveys voicing and manner (Summerfield, 1987). The McGurk effect (McGurk and MacDonald, 1976) that occurs when audition and vision provide incongruent tokens illustrates AV integration. For example, when presented with visual velar /ka/ and auditory bilabial /pa/, normally hearing individuals tend to report the illusory fusion alveo-dental /ta/.

Place of articulation is acoustically conveyed by formant transitions, more precisely by the second and third formants, located in high frequencies. The perception of place of articulation is difficult when the acoustic signal is masked by noise (Miller and Nicely, 1955), but is well improved when visual speech cues are added to the signal. When speakers produce /apa/, /ata/, or /aka/, the place of articulation is visually distinguishable by the listener by virtue of the lip movements. Visual information from a talker's face can facilitate speech perception when the environment is less than optimal (Sumby and Pollack, 1954; MacLeod and Summerfield, 1987) or when the listener is hearing impaired (Erber, 1972; Huyse et al., 2012).

Surprisingly, the effect of visual information on speech perception in noise by children with SLI has been little studied up to now. As children with SLI demonstrated a deficit in auditory categorical perception of place of articulation feature (Sussman, 1993; Ziegler et al., 2005; Gerrits and de Bree, 2009), they might take advantage of visual cues, maybe to a greater extent than children with TLD. A few studies examined this question. It appeared that visual articulatory cues influenced adults and children with language impairment to a lesser extend than participants with TLD (Ramirez and Mann, 2005; Norrix et al., 2007; Leybaert and Colin, 2008; Meronen et al., 2013). Ramirez and Mann (2005) compared adults with dyslexia and with auditory neuropathy (AN) to adults with TLD. Participants were presented with natural speech stimuli that were masked with speech-shaped noise at various intensities, in an auditory only (AO), or in an audio-visual (AV) condition. Noise masked the perception of stimuli in AO more in dyslexic and AN participants than in participants with TLD. Patients with AN benefitted from the pairing of visual articulatory cues to auditory stimuli, indicating that their speech perception impairment reflects a peripheral auditory disorder. In contrast, dyslexic participants showed less effective use of visual articulatory cues in identifying masked speech stimuli as well as a lower speechreading capacity relative to control participants. To sum up, language impairment extends beyond the AO modality, and participants with language problems (here: dyslexics) have impoverished AV perception, due to their deficit in speechreading abilities (see also Blau et al., 2010, for a discussion about letter-speech sound integration in developmental dyslexia).

Norrix et al. (2007) presented pre-school children with TLD and with SLI with three syllables /bi/, /di/ and /gi/ in AO, AV congruent and AV incongruent McGurk stimuli (A/bi/ V/gi/ for example). Speechreading ability was not measured. Both groups were at ceiling when asked to identify tokens in AO and AV congruent modalities. A stronger McGurk effect was found for the TLD group compared to the SLI group, indicating that children with SLI were less impacted by the processing of visual speech cues.

Leybaert and Colin (2008) presented French-speaking SLI and TDL children matched for chronological age with video clips of a man speaking /bi/ and /gi/, in optimal listening conditions (no noise). Children with SLI were less likely than TLD children to correctly identify /bi/ and /gi/ syllables in AO as well as in VO modalities. Children with SLI also showed a smaller visual gain (VG), as measured as the improvement of accuracy between AO and AV congruent conditions. When perceiving McGurk incongruent stimuli (e.g., A/gi/V/bi), children with SLI reported more auditory-based responses, fewer visually based responses and fewer combination responses than children with TLD. To sum up, when auditory information is contradicted by visual information such as in McGurk stimuli, children with SLI are less influenced by visual information than children with TLD.

In a recent paper, Meronen et al. (2013) investigated the effect of signal-to-noise ratio (SNR) on the perception of audiovisual speech in 8-year-old children with developmental language disorder and a sample of children with TLD. Performance was measured for /apa/, /ata/, /aka/ presented in AO modality, VO modality, and in AV incongruent (A/p/ V/k/). Three sound intensities (24, 36, and 48 dB) and noise levels (−12, 0, and +6 dB) were used. Both groups achieved similar performance in the AO condition, but children with developmental language disorders reached lower performances than children with TLD in the VO modality. In response to McGurk stimuli, children with developmental language disorders showed more auditory /p/ responses and less visual /k/ responses than children with TLD. In addition, SNR significantly impacted the proportion of auditory and visual responses in children with TLD, who gave more visual responses when the SNR was more adverse. In contrast, the pattern of responses of children with developmental language disorders was not influenced by SNR. To sum up, the less accurate recognition of visual speech can explain the weaker McGurk effect in the children with developmental language disorders, as well as the lack of impact of SNR on their pattern of auditory and visual responses. This conclusion is in agreement with Norrix et al. (2007) and Leybaert and Colin (2008).

In the current study, we extended the previous investigation by examining the impact of visual cues in the context of a *masking release paradigm*, in school aged children with and without SLI. The release from masking phenomenon refers to the fact that listeners presented with syllables embedded in noise show increased speech intelligibility in fluctuating noise (i.e., modulated in amplitude) compared to stationary noise (Nelson et al., 2003; Füllgrabe et al., 2006). This is an adaptative mechanism since many natural background noises are temporally fluctuating (e.g., surrounding conversations). The masking release phenomenon suggests that listeners are able to “listen in the noise dips” that is, in short temporal minima present in fluctuating noise but absent in stationary noise.

Although children with SLI have lower performances in perceiving auditory syllables masked by either stationary or fluctuating noise, they show an auditory masking release effect comparable to children with TLD (Ziegler et al., 2005). In the present study, we used a new audio-visual masking release paradigm, in which 6 consonants (/apa/, /afa/, /ata/, /asa/, /a∫a/, /aka/) were presented in AO, in VO, and in AV conditions. All stimuli were covered either by stationary or fluctuating noise. AV stimuli were either congruent (e.g., A/apa/ V/apa/) or incongruent (e.g., A/apa/ V/aka/). In previous studies, adults and children with TLD showed larger visual gains when the syllables were masked by stationary noise than when they were masked by fluctuating noise. For incongruent AV stimuli, they gave a majority of visually-based responses when syllables were masked by stationary noise, and more fusions and auditory-based responses when syllables were presented with fluctuating noise (Huyse et al., 2012; Huyse et al., in revision).

As in our previous research, we expected to observe a strengthening of the McGurk effect with fluctuating noise compared to stationary noise in children with TLD. Our main interest was to test whether children with SLI would also show a strengthening of the McGurk effect with fluctuating noise, meaning that their performance would approach that of the TLD children in the conditions of an auditory masking release. We used two types of McGurk stimuli: the plosives A/apa/ V/aka/, giving rise to the fusion /ata/, and the fricatives A/afa/ V/a∫a/, leading to the fusion /asa/ (Berthommier, 2001). The interest of A/afa/ V/a∫a/ is that a dominance of the video responses / ∫ / is observed (Berthommier, 2001; Huyse et al., 2012). If children with SLI recognize /a∫a/ in VO condition, their responses to A/afa/V/a∫a/ would show clear influence of visual information, as it is the case in the TLD children.

Unisensory auditory (AO stimuli) and lipreading (VO stimuli) performances, as well as audio-visual speech perception (AV congruent stimuli) were also measured. In AO, we expected a larger speech-in-noise deficit in children with SLI compared to TLD children, but a similar masking release effect in both groups (Ziegler et al., 2005). In VO, children with SLI would experience more difficulties than TLD children. The visual gain measures the improvement of speech identification in AV compared to AO, due to efficient use of visual cues to recover place of articulation and manner features. Compared to children with TLD, children with SLI would experience less influence of visual cues, and a reduced visual gain.

These hypotheses were tested in two experiments. In Experiment 1, six voiceless consonants were presented in a /aCa/ context, masked by either stationary or amplitude modulated noise (8 Hz and 128 Hz). The stimuli were presented in Audio-only (AO), Visual Only (VO), and Audio-visual (AV) congruent and AV incongruent conditions.

In Experiment 2, larger groups of children with SLI and children with TLD were recruited. Twelve consonants (six voiceless and six voiced) masked either by stationary or by amplitude modulated noise (at 8 Hz) noise were presented in AO, VO, AV congruent conditions, and four McGurk stimuli (two with plosives, two with fricatives) were used. The first aim of Experiment 2 was to replicate the results of Experiment 1 with a large set of consonants. The second aim was to evaluate the specific reception of voicing, place and manner by information transmission (IT) analyses performed on the basis of confusion matrices (Miller and Nicely, 1955). Specifically, we expected an increase of IT in AV compared to AO for the reception of manner and place of articulation, but not for voicing, which has no visible correlate. For the same reason, the percent of IT would be higher than 50% for manner and place of articulation in VO, but around 50% for voicing. Compared to children with TLD, we expected to observe a lower percent of IT across the three features in children with SLI, with a possible enhanced deficit for place of articulation.

## Experiment 1

### Material and method

#### Participants

Fifteen French-speaking children with SLI (8 boys) were recruited in special language classes and through an association of parents of children with SLI. The participants met the following criteria: (1) presence of a long-lasting and severe impairment of expressive and/or receptive language, diagnosed as SLI by a neuro-pediatrician in a multi-diciplinary team; (2) no history of hearing loss and no malformation of speech organs; (3) a score > 132 points on the pragmatic component (scales C to G) of the Children's Communication Checklist (Bishop, 1998); (4) a non-verbal IQ > 85 on the French version of the Wechsler Intelligence Scale for children (Wechsler, 1996); and (e) at least 1.5 SD below the age-appropriate mean on the three language tests described below. One child was excluded from our sample, due to the absence of a recent assessment of persistant language impairment. The final sample included 14 children (7 boys) ranging in age from 8 years 7 months to 14 years 5 months (mean age: 138 months; *SD* = 25 months). All children had measured reading and spelling levels corresponding at least to the end of first grade.

Language assessment tests included: (a) reading aloud of pseudowords and phonically regular and irregular frequent words of the Odedys Test (Jacquier-Roux et al., 2005); (b) Repetition of Difficult Words from the L2MA (Chevrie-Muller et al., 1997); (c) receptive lexical knowledge (EVIP, French version of the PPVT, (Dunn et al., 1993): children have to listen to a word said by the experimenter and to designate the picture corresponding to that word, among four pictures.

A control group of French-speaking children with TLD was recruited. None of them had any history of language or hearing disorders or used hearing aids. Each child with TLD was matched with a child with SLI, based on chronological age and gender. The control group included 14 children (7 boys) ranging in age from 9 years 1 months to 14 years 6 months (mean age: 141 months; SD 25 months). The scores of the children with TLD were within normal limits for the three language tests.

The characteristics of the participants and a summary of the language test scores of the children with SLI and those with TLD are found in Table 1. All participants had normal or corrected-to-normal vision and none of them reported any difficulties with viewing the visual stimuli presented in this study.

**Table 1:** **Characteristics of children with SLI and of TLD controls—Experiment 1**.

	**SLI**	**TLD**	**Group effect *F*_(1, 26)_ = *p*-value**
Age in years, months (range)	11.6	11.9 (9.1–14.6)	*Ns*
	(8.7–14.5)	(9.1–14.6)	
Word repetition (*SD*)	20.07	29.86	*F* = 54.02
	(4.97)	(0.36)	*p* < 0.001
Vocabulary EVIP (*SD*)	91.14	132.71	*F* = 29.81
	(22.65)	(17.28)	*p* < 0.001
Irregular words (*SD*)	7.71	19.07	*F* = 37.63
	(6.68)	(1.82)	*p* < 0.001
Regular words (*SD*)	10.29	19.86	*F* = 28.62
	(6.67)	(0.53)	*p* < 0.001
Pseudo words (*SD*)	7.07	17.50	*F* = 40.34
	(5.89)	(1.74)	*p* < 0.001

Word repetition values indicate number of correct responses (out of 30) on the repetition test taken from the L2MA language battery; Values for Vocabulary indicate raw score on the EVIP test; Irregular Words, Regular Words and Pseudo Words values indicate number of correct responses (out of 20) on the reading tests for frequent items taken from Odedys battery. Standard deviations are in brackets.

The project has been reviewed and approved by the University research ethic board. Informed consent was obtained from the parents of all participants, and children provided a verbal acceptance prior to their participation. They were informed that they could interrupt their participation if they felt any problem during the experiment.

#### Stimuli

Stimuli were composed of vowel-consonant-vowel (VCV) syllables with the consonants /p, t, k, s, f, ∫/ interposed between two /a/ vowels. A male speaker of French was videotaped while saying these syllables. He was filmed from the bottom of the nose to the chin. The production of each stimulus began and ended in a neutral position, with the mouth closed. Videos (Quicktime movie files, 21 by 21 cm) were displayed centered on a 15-inch MacBook Pro laptop on a black background. Three productions of each /aCa/ stimulus were digitally recorded and audio tracks were equalized in level. Eighteen stimuli (six syllabes × three repetitions) were used to create the AV, AO and VO trials. Stimuli were delivered through Sennheinser HD 121 Pro headphones.

The congruent AV stimuli included digital audio-video files of the speaker saying and articulating the /aCa/ stimuli. For the AO condition, an image of the speaker, appearing neutral and with mouth closed was presented along with the auditory stimulus. For the VO condition, the audio was turned off. Finally, incongruent AV McGurk stimuli were created by carefully combining audio files with non-corresponding video files and matching their onset. We used three repetitions of the two following stimuli: audio /apa/ with visual /aka/ (fusion /ata/) and audio /afa/ with visual /a∫a/ (fusion /asa/).

The total number of items was 180 stimuli (six syllables × three repetitions × three modalities × three types of noise +18 McGurk stimuli, randomly mixed). Four blocks of 45 items were constructed. In each block, the order of appearance of the stimuli was fixed and identical for all participants.

***Auditory noise.*** Each signal was digitalized at a 22,050 Hz sampling frequency. Throughout all conditions of the experiment, stimuli were embedded in noise which was either stationary (i.e., unmodulated), either modulated in amplitude. Modulation in amplitude was achieved by using a white Gaussian noise low-pass filtered at 500 Hz (WGNf). The expression describing the sine-wave modulator, *m*(*t*), was

$$m(t)=[1+\cos{\left(2\pi f_{m}t\right)}^{*}WGNf]$$

where the 1st-order modulation frequency *f*_*m*_ was 8 and 128 Hz. The noise was then added to the signal. The SNR was fixed at −23 dB (prior to the 500 Hz filtering). This SNR was determined in a preliminary experiment so as to yield a consonant identification performance of about 40% correct under stationary noise (in AO condition).

#### Procedure

The experiment was conducted in a dimly-lit quiet room. Participants were seated in front of the laptop and fitted with headphones. Stimuli were presented on a monitor positioned at eye level, 70 cm from the participant's head. Participants were given verbal and written instructions to watch the computer monitor and listen for speech sounds that would be heard over headphones. They were informed about the identity of the six syllables that would be presented. They were instructed that for some trials, there would be a speech sound but the face would not move (i.e., the AO stimuli), sometimes the face would move but there would be no speech sound (the VO stimuli) and sometimes there is a speech sound and a moving face (i.e., the AV stimuli). No information was given about the presence of the McGurk incongruent stimuli.

Participants were instructed to designate a letter corresponding to the consonant they thought the speaker had said. The six letters were taken from a speech therapist kit (*La planète des Alphas*, Huguenin and Dubois, 2006) which was unknown both from the children with SLI and the children with TLD. Sometimes, children also spontaneously repeated the syllable aloud. Their responses were recorded by the experimenter. They were given 20 practice trials, including AO, VO, and AV congruent stimuli, during which they were provided with feedback regarding the correct responses. Prior to beginning the experimental trials, they were informed that they would no longer receive any feedback.

Following the practice session, participants were presented with the four experimental blocks. The sequence of presentation of the blocks was counterbalanced across participants. After the four experimental blocks, they were given a block of 54 stimuli presented without noise. This quiet block consisted of the six syllables × three repetitions × three modalities (AO, VO, and AV congruent). In a second session, they were submitted to the three language tests.

Participant's percent-correct identification of the VCV syllables presented in each of these conditions served as the dependent measure. For McGurk stimuli, the percent responses corresponding to Audio, Visual and Fusion responses were recorded.

The experiment took place in two 30 min sessions. The first session was devoted to the collection of language measures, and the second one to the experimental data. The experimenter was careful about the attention and concentration of the children, and proposed breaks if necessary.

### Results

Results in noise modulated at 8 Hz and noise modulate at 128 Hz were averaged for more clarity and because they were not significantly different.

#### Single modality conditions

First, results were analyzed in the AO modality in order to ascertain whether our experimental design generated a masking release effect, i.e., higher performances in AM noise than in ST noise. The percentage of correct identification of children with SLI and with TLD for quiet, AM noise, and ST noise, and the masking release effect are presented in Table 2. A clear masking release effect was observed for both groups: performance was about 30% better in AM noise than in ST noise.

**Table 2:** **Mean percent correct responses for AO in quiet, AM noise and ST noise, and mean value for the masking release effect**.

	**SLI**	**TLD**
Silence	97.2 (8.9)	100
AM noise	76.2 (11.4)	85.4 (8.2)
ST noise	47.6 (9.2)	52.3 (10.4)
Masking release	28.6 (10.1)	33.0 (12.9)

Standard deviations are in brackets.

An ANOVA with repeated measures on Noise (3 levels: quiet, AM, and ST) and Group (children with SLI, children with TLD) was run on these data. The analysis yielded a significant effect of Noise, [*F*_(2, 52)_ = 327.94, *p* < 0.001], and of Group, [*F*_(1, 26)_ = 4.94, *p* < 0.05]. The Group × Noise interaction was not significant. Orthogonal contrasts were made on the effect of Noise. The first contrast, comparing the results in quiet on the one hand, and in AM and ST Noise on the other hand, was highly significant, [*F*_(1, 26)_ = 566.51, *p* < 0.001]. The second contrast, comparing the results in AM noise and ST noise, was highly significant too, [*F*_(1, 26)_ = 198.27, *p* < 0.001]. None of these contrasts interacted with the Group effect. To sum up, performance was better in modulated noise than in stationary noise, and better in quiet than in noisy conditions. The 4.4% difference of masking release between children with SLI and TLD was not significant.

Second, results were analyzed in the VO modality. As expected, children with TDL achieved better performances in VO than children with SLI, regardless of whether the stimuli were presented in quiet, in AM noise, or in ST noise (see Table 3). These data were entered in a repeated measures ANOVA, with Group as between subjects factor, and Noise (3 levels: quiet, AM, and ST) as within subjects factor. Only the Group effect was significant, [*F*_(1, 26)_ = 16.86, *p* < 0.001]. Neither the Effect of Noise, nor the Group × Noise interactions were significant. To sum up, children with SLI achieved lower performance in identification of syllables presented in speechreading; as expected, auditory noise had no significant effect on the performance in VO.

**Table 3:** **Mean percent correct responses for VO in quiet, AM noise and ST noise**.

	**SLI**	**TLD**
Silence	54.4 (17.4)	74.5 (7.8)
AM noise	56.7 (12.4)	69.4 (8.0)
ST noise	56.8 (12.4)	71.0 (9.3)

Standard deviations are in brackets.

#### Congruent AV modality (AV)

Percentages of correct identification of children with SLI and of children with TLD for AV in quiet, in AM noise, and in ST noise are presented in Table 4. The performance of children with SLI was significantly lower than the performance of children with TLD in all three conditions.

**Table 4:** **Mean percent correct responses for AV in quiet, in AM noise, and ST noise, and mean value for Visual Gains (VG)**.

	**SLI**	**TLD**
AV (quiet)	97.6 (3.6)	100
AV/AM	89.1 (10.2)	95.3 (4.7)
AV/ST	83.8 (9.8)	91.3 (6.5)
VG/AM	56.0 (36.7)	61.2 (50.2)
VG/ST	69.1 (17.7)	81.3 (15.3)

Standard deviations are in brackets.

A repeated measures ANOVA with Noise (3 levels: quiet, AM, and ST) as within-subjects factor and Group (children with SLI, children with TLD) as between-subjects factor was run on these data. The analysis yielded significant effects of Noise, [*F*_(2, 52)_ = 31.58, *p* < 0.001], and of Group, [*F*_(1, 26)_ = 7.35, *p* < 0.05]. The Group × Noise interaction was not significant. Orthogonal contrasts were made on the effect of Noise. The first contrast, comparing the results in quiet on the one hand, and in AM and ST Noise on the other hand, was highly significant, [*F*_(1, 26)_ = 55.08, *p* < 0.001]. The second contrast, comparing the results in AM noise and ST noise, was highly significant too, [*F*_(1, 26)_ = 10.19, *p* < 0.005]. None of these contrasts interacted with the Group effect.

We calculated the visual gains (VG) in both groups. Visual gain refers to relative increase in AV speech perception performance due to the addition of visual information to the auditory signal (Sumby and Pollack, 1954). We computed VG in ST and AM noise using the following formula:

$$\begin{array}{l} \text{VG}/\text{ST }=\;(\text{AVST}-\text{AOST})/(\text{100}-\text{AOST})\\ \text{VG}/\text{AM }=\;(\text{AVAM}-\text{AOAM})/(\text{100}-\text{AOAM}) \end{array}$$

The values of the VG are displayed in Table 4. An ANOVA with repeated measures on Noise and Group as between subjects factor yielded no effect of Noise, Group and no interaction.

Overall, the data showed that children with SLI had lower performance on AV syllable identification than children with TLD. However, children with SLI did not differ from children with TLD in masking release effect, nor in visual gain.

#### McGurk effect

**Figure 1:**
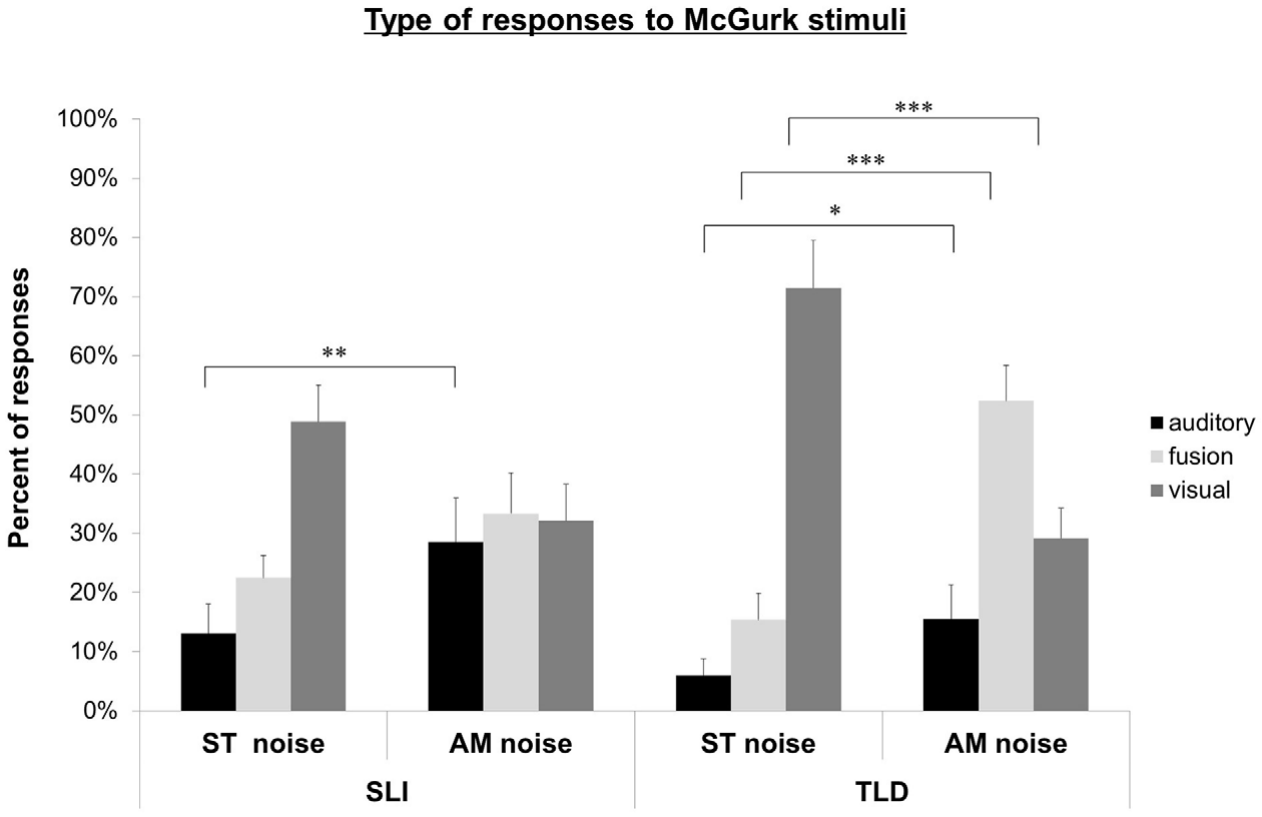
**Experiment 1**. Auditory, fusion, and visual responses to McGurk stimuli for SLI and TLD groups in ST and AM noise conditions. ^*^p < 0.05; ^**^p < 0.01; ^***^p < 0.001.

The percentages of auditory, visual and fusion responses were computed relative to the total amount of responses to McGurk stimuli. The distribution of responses is shown in Figure 1 for children with SLI and TLD children. First, the response pattern of each group was examined to evaluate the impact of noise condition (ST vs. AM) on AV speech integration. Second, the groups were compared in order to examine the effect of language impairment.

In ST noise, children with TLD mainly gave a low rate of auditory responses (5.9%; SD: 10.6), and fusion responses (15.4%; SD: 16.5), and a high rate of visual responses (71.5%; SD: 30.3). Compared to ST noise, children with TLD gave significantly more auditory responses [15.5%; SD: 21.9; *F*_(1, 13)_ = 6.63, *p* < 0.05], a higher number of fusion responses [52.4%; SD: 22.6; *F*_(1, 13)_ = 20.2, *p* = 0.001], and significantly less visually responses [29.1%; SD: 19.3; *F*_(1, 13)_ = 39.97, *p* < 0.001] in AM noise.

In ST noise, children with SLI gave 13.0% (SD: 18.7) of auditory responses, 22.5% (SD: 14.0) of fusion responses, and 48.9% (SD: 23.2) of visual responses. In AM noise, they gave more auditory responses [28.5%; SD: 27.9; *F*_(1, 13)_ = 13.76, *p* < 0.005] than in ST noise. The percent of fusion responses (33.3%; SD: 25.6) and of visual responses (32.1%; SD: 23.0) was not significantly different from that in ST noise.

Compared to children with TLD, children with SLI had a lower rate of visual responses in ST noise, *F*_(1, 26)_ = 4.92, *p* < 0.05, and less fusions in AM noise, *F*_(1, 26)_ = 4.36, *p* < 0.05. No other difference was significant.

To sum up, the pattern of responses to McGurk stimuli was clearly modified by the degree of degradation of auditory information in children with TLD: AM noise decreased the rate of visual responses, and increased auditory, and fusion responses. For children with SLI, AM noise increased auditory responses, confirming children's intact auditory masking release effect; however, AM noise has no impact for fusion and visual responses, coherently with SLI's deficit in processing visual speech information.

### Discussion

The present study examined the impact of SLI on AV speech perception with a masking release paradigm, already used to study audiovisual integration in TLD children and children with cochlear implants (Huyse et al., 2012). Several results are to be emphasized, in relation to our predictions. First, in AO modality, children with SLI showed a deficit in consonant perception presented in quiet, stationary noise or modulated noise. Despite their speech-in-noise deficit, children with SLI experienced a clear masking release effect, which was not significantly different from that of TLD children: their speech intelligibility was increased in the modulated noise compared to the stationary noise (Ziegler et al., 2005). The average size of the effect was around 30%, which is relatively high compared to the 10% found by Ziegler et al. (2005). Difference between the SNR used in these two studies could be an explanation. Ziegler et al. (2005) used a SNR of 0 dB so as to yield an auditory performance of approximately 50% correct with ST noise. We wanted to obtain a lower level of correct responses in AO/ST, in order to observe both an auditory masking release effect and a visual gain, and we used a SNR of −23 dB. With a lower rate of correct responses in AO/ST as a baseline, it is easier to obtain larger masking release values.

Second, in VO modality, children with SLI were less accurate than TLD children in identification of the six consonants belonging to different visemes. This result is coherent with the notion that children with SLI experience difficulties in perceiving place of articulation (Sussman, 1993; Gerrits and de Bree, 2009), and reveals that this deficit is not specific to auditory processing but could be extended to visual processing (Meronen et al., 2013).

Third, children with SLI performed less well than TLD children in AV congruent modality, indicating that the difficulty of processing of acoustic cues in the AO modality, also impacted audio-visual processing. Surprisingly, the visual gains of children with SLI did not significantly differ from those of the control group.

Fourth, children with TLD were clearly influenced by the degree of degradation of auditory information in AV incongruent modality. In ST noise, when little auditory information is available, participants with TLD mainly relied on visual information. When the speech signal is more available thanks to the existence of noise dips (AM noise), participants with TLD increased their number of auditory responses and their number of fusions even more impressively, while their number of visually-based responses decreased. To sum up, when both auditory and visual information are available (as in AM noise), and participants are able to process them (as are children with TLD), conditions needed to generate McGurk fusions are met. The response pattern of children with SLI to McGurk stimuli was different from that of children with TLD, and coherent with their lower speechreading skills in VO. In ST noise, they gave less visual responses than children with TLD, and in AM noise they reported less fusions than children with TLD. The McGurk effect for the classical pair A/p/V/k/, characterized by a backward shift of the percept from /p/ to /t/ does not work for them to the same degree as for children with TLD. These observations indicate a smaller influence of the visual speech cues on their speech perception processes.

Taken together, the results of Experiment 1 confirm that the difficulties in building accurate phonemic categories is not limited to the auditory modality but is supra-modal in children with SLI. This deficit appears in their responses to stimuli in VO condition, but also to McGurk stimuli. However, the redundancy between visual and auditory information helps children with SLI, as indicated by their visual gain not different from that of children with TLD. Therefore, a more in-depth analysis of how children with SLI process manner, voicing and place of articulation seems necessary in order to get a clearer picture.

Limits of Experiment 1 are the reduced sample of children with language impairment, as well as the number of stimuli used to evaluate the McGurk effect. Therefore, we carried out a second experiment, using a larger set of stimuli. In order to better compare the use of phonetic cues by children with SLI and children with TLD, we also computed the percent of information transmitted for place of articulation, manner, and voicing in AO, AV, and VO.

## Experiment 2

Experiment 2 aimed at generalizing the outcomes of Experiment 1, on a new and larger sample of participants. We introduced several changes in our methodology in order to better evaluate the use of visual information by children with SLI. We included six voiceless and six voiced consonants corresponding to the six visemes used in Experiment 1. Auditory, fusion and visual responses given to McGurk stimuli were measured separately for plosive stimuli A/apa/V/aka/ and A/aba/V/aga/, and fricative stimuli A/afa/V/a∫a/ and A/ava/V/aja/. The interest of the fricative stimuli is that a dominance of the visual responses /∫/ or /j/ is observed (Berthommier, 2001; Huyse et al., 2012). If children with SLI recognize /a∫a/ and /aja/ in VO condition, their responses in incongruent AV would show clear influence of visual information, as it is the case in the TLD children. In order to maintain the duration of testing in a reasonable amount of time, only ST noise and AM noise at 8 Hz were used.

In addition to measuring the performance for AO, VO, AV congruent and incongruent stimuli, we computed the specific reception of phonetic features (voicing, place, and manner) by analyses of information transmission (IT) (Miller and Nicely, 1955). Analyses of IT in auditory recognition of speech-in-noise have revealed that children with SLI have a deficit in place, manner, and even more in voicing perception (Ziegler et al., 2005). The present study will allow us to extend these results by examining IT for place, manner and voicing features, in AO, VO, and AV modalities.

We recruited new and larger groups of children with SLI and TDL children whose language performances were examined in a more detailed way (as in Ziegler et al., 2005). We systematically proposed all children to name aloud the syllables in Experiment 2.

### Material and method

#### Participants

Fifty-four children, all native and monolingual speakers of French, were recruited as participants. Twenty-seven children (13 boys and 14 girls) constituted the TLD group, and 27 children (17 boys and 10 girls) constituted the group of children with SLI. The two groups were matched as closely as possible by gender, chronological age and by score at the Raven matrices intelligence test (Raven, Court and Raven, 1998). The mean age was 10 years 8 months (range: from 7 years 4 months to 12 years 9 months) for the children with SLI, and 10 years 2 months (from 7 years 6 months to 13 years 8 months) for the TLD children (see Table 5). In order to include a child as a participant with SLI, he/she had to present the characteristics outlined in the methodology of Experiment 1.

**Table 5:** **Characteristics of children with SLI and TLD controls in Experiment 2**.

	**SLI**	**TLD**	**Group effect ***F***_**(1, 26)**_ = *p*-value**
Age in years, months (range)	10.9	10.2	*Ns*
	(7.4–12. 9)	(7.6–13.9)	
Raven (*SD*)	28.44	30.37	*F* = 4.06
	(4.24)	(3.56)	*p* < 0.05
EVIP (*SD*)	89.15	116.81	*F* = 14.25
	(26.91)	(26.95)	*p* < 0.001
Morpho-syntax (*SD*)	14.29	6.00	*F* = 33.94
	(6.06)	(4.24)	*p* < 0.001
Word repetition (*SD*)	15.74	28.11	*F* = 122.66
	(5.51)	(1.84)	*p* < 0.001
Irregul. words (*SD*)	8.67	18.19	*F* = 74.82
	(5.37)	(1.96)	*p* < 0.001
Regular words (*SD*)	12.03	19.41	*F* = 47.11
	(5.48)	(1.05)	*p* < 0.001
Pseudo words (*SD*)	8.15	17.26	*F* = 79.76
	(4.89)	(2.03)	*p* < 0.001

Values for Vocabulary indicate raw score on the EVIP, the French version of the PPVT test; Morpho-syntax indicates the number of errors on the ECOSSE picture/sentence word comprehension test. Word repetition values indicate number of correct responses (out of 30) on a sub-test taken from the L2MA language battery. Irregular Words, Regular Words and Pseudo Words values indicate number of correct responses (out of 20) on the reading tests for frequent items taken from Odedys battery.

Hearing and visual abilities of the children with TLD were assessed through a questionnaire filled in by their parents. Children whose parents reported a hearing acuity problem, or who were followed in speech therapy, were removed from the sample.

All children were submitted to the Progressive Matrices Color Raven test (Raven et al., 1998). Language assessment tests included: (a) receptive lexical knowledge (EVIP, French version of the PPVT, Dunn et al., 1993); (b) a standardized test of morpho-syntax, l'E.CO.S.SE (French version of the TROG test, Lecocq, 1996), and (c) Repetition of Difficult Words from the L2MA (Chevrie-Muller et al., 1997). All TLD children presented results comprised between −1.5 SD and +1.5 SD to the three language tests. Reading assessment involved reading aloud Regular and Irregular frequent words, and Pseudowords from the battery Odedys-2 (Jacquier-Roux et al., 2005). The characteristics of the participants and a summary of the language test results are found in Table 5.

The project has been reviewed and approved by the University research ethic board. Informed consent was obtained from the parents of the participants, and children provided a verbal acceptance prior to their participation. They were informed that they could interrupt their participation if they felt any problem during the experiment.

#### Stimuli

Movie files of digital AV stimuli were extracted from the same database as those of Experiment 1: /apa/, /afa/, /ata/, /asa/, /aka/, /a∫a/, /aba/, /ava/, /ada/, /aza/, /aga/, and /aӡa/. Three productions of each /aCa/ stimulus were used. The AV (congruent and incongruent), AO, and VO stimuli were constructed in the same way as in Experiment 1. We used four different AV incongruent McGurk stimuli. Two were the classical stimuli with plosive consonants: A/apa/ V/aka/ (→fusion /ata/), and the A/aba/ V/aga/ (→ fusion /ada/). The other two were new combinations based on the fricative pairs: A/afa/ V/a∫a/ (→ fusion /asa/) and A/ava/ V/aӡa/ (fusion /aza/) (Berthommier, 2001). As the recognition of /∫/ and /ӡ/ are generally good in speechreading, these fricative pairs offer a new opportunity to examine the processing of visual speech cues by children with SLI.

The AO, VO, and AV stimuli were presented masked by either stationary noise (ST, i.e., unmodulated), or amplitude modulated noise (AM at 8 Hz). The SNR was fixed at −23 dB.

The total amount of items was 252 stimuli (12 syllables × 3 repetitions × 3 modalities × 2 types of noise + 36 McGurk stimuli) randomly mixed and divided in four blocks. In each block, the presentation order of the stimuli was fixed and similar for all participants. In addition, a last bloc containing 120 stimuli (12 syllables × 3 repetitions, × 3 modalities + 12 McGurk stimuli) was presented in quiet, i.e., without noise.

#### Procedure

The procedure was the same as in Experiment 1, except that participants were instructed to answer by verbally repeating the syllable they perceived. Verbal repetition is an immediate response and is resistant to decay from phonological short-term memory. When the understanding of the syllable was difficult because of articulatory problems, children were encouraged to use a lexical evocation: for example, a child perceiving correctly the syllable /aka/ but pronouncing it /ata/, said I heard /ata/ as in /tamj$\tilde{ͻ}$/—the real pronunciation of this word is /kamj$\tilde{ͻ}$/(truck in English). A series of 12 pictures, beginning with the 12 consonants, was prepared to help children to answer. The experimenter recorded the responses. The stimuli in the practice session were representative of the conditions participants would experience in the actual experimental trials, except the McGurk stimuli. For practice trials, subjects were provided with feedback regarding the correct responses. Before beginning the experimental trials, subjects were told that they would no longer receive any feedback.

Following practice, participants were presented with the four experimental blocks. The order of the blocks was counterbalanced across participants. After the four experimental blocks, participants were given a block of 120 stimuli presented in quiet. In a second session, participants were submitted to the Raven matrices, the language and the reading tests.

Participant's percent-correct identification of the syllables presented in each of these conditions served as dependent measure. For McGurk stimuli, we recorded the percent of Auditory, Visual, and Fusion responses.

### Results

#### Single modality conditions

The percentage of correct identification of children with SLI and of the TLD children for stimuli in quiet, AM noise, and ST noise in the AO modality is presented in Table 6. Visual inspection of the data revealed that the children with SLI differed from the TLD children in the three conditions. A masking release effect was observed: performance was about 35% better in AM noise than in ST noise for children with SLI, and 39% for children with TLD.

**Table 6:** **Mean percent correct responses for AO in quiet, AM noise and ST noise, and mean values for the masking release effect**.

	**SLI**	**TLD**
Quiet	94.65 (6.11)	98.87 (1.77)
AM noise	51.65 (9.87)	61.52 (7.17)
ST noise	16.56 (7.76)	22.63 (6.20)
Masking release	35.08 (9.47)	38.89 (7.47)

Standard deviations are in brackets.

The data were entered in an ANOVA with Noise (quiet, AM, and ST) as within-subjects factor and Group as between-subjects factor. The analysis yielded significant effects of Noise, *F*_(2, 104)_ = 2154.92, *p* < 0.001, and Group, *F*_(1, 52)_ = 26.37, *p* < 0.001. The Noise × Group interaction was just below significance level, *F*_(1, 104)_ = 3.01, *p* = 0.054. The data corresponding to the masking release effect were analyzed with a separate ANOVA, with Group as between-factor: no effect of Group was found (*p* = 0.11). To sum up, children with SLI achieved poorer recognition of auditory speech, but a similar masking release effect as children with TLD.

The percentage of correct identification for stimuli in VO in quiet, AM noise and ST noise is presented in Table 7. Children with TLD better identified stimuli in the three conditions than children with SLI. The percent of correct responses for VO stimuli was entered in a repeated measures ANOVA with Noise (quiet, AM noise and ST noise) as within subjects factor, and Group as between subjects factor. The analysis yielded a significant effect of Group, *F*_(1, 52)_ = 7.69, *p* < 0.01; and of Noise, *F*_(2, 104)_ = 5.24, *p* < 0.01. No interaction was found. To sum up, children with SLI had poorer lipreading performance than children with TLD.

**Table 7:** **Mean percent correct responses for VO in quiet, AM noise and ST noise**.

	**SLI**	**TLD**
Quiet	28.09 (9.72)	34.88 (9.60)
AM noise	26.85 (8.95)	33.85 (7.90)
ST noise	26.03 (8.69)	30.04 (9.65)

Standard deviations are in brackets.

#### Congruent AV modality (AV)

The percentages of correct identification of children with SLI and of TLD children for AV in quiet, AM noise, and ST noise are presented in Table 8. The performance of children with SLI was lower than that of TLD children in the three conditions. The data were entered in a repeated measures ANOVA with Group as between subjects factor and Noise (Quiet, AM Noise, ST Noise) as within subjects factor. The analysis yielded a significant effects of Group, *F*_(1, 52)_ = 33.41, *p* < 0.001, and Noise, *F*_(2, 104)_ = 1.060, *p* < 0.001. The interaction between Group and Noise was also significant, *F*_(2, 104)_ = 6.76, *p* < 0.005. The effect of Noise was further analyzed with two orthogonal contrasts. The first one, comparing the performance in Quiet to the mean performance for AM and ST noise, was highly significant, *F*_(1, 52)_ = 1589, *p* < 0.001, as was the interaction with Group, *F*_(1, 52)_ = 11.95, *p* < 0.005. The second contrast, comparing performance in AM noise and in ST noise was highly significant, *F*_(1, 52)_ = 394.05 *p* < 0.001, and did not interact with Group. To sum up, the effect of Noise on AV speech perception was larger in children with SLI than in children with TLD.

**Table 8:** **Mean percent correct responses for AV in quiet, AM noise and ST noise, and mean value for Visual Gains (VG)**.

	**SLI**	**TLD**
AV/Quiet	96.91 (5.57)	99.38 (1.78)
AV/AM	64.40 (7.10)	74.07 (7.22)
AV/ST noise	44.96 (7.35)	52.57 (7.02)
VG/AM	25.33 (13.11)	32.11 (15.98)
VG/ST	33.71 (8.74)	39.92 (9.42)

Standard deviations are in brackets.

We computed VG/ST and VG/AM noise using the same formula as in Experiment 1. A repeated measures ANOVA with Noise as within subjects factor, and Group as between subjects factor yielded significant effects of Noise, *F*_(1, 52)_ = 14.46, *p* < 0.001, and of Group, *F*_(1, 52)_ = 6.54, *p* < 0.05. The Group × Noise interaction was not significant. To sum up, children with SLI had lower standardized VG than TLD children, both in ST and AM Noise.

#### McGurk effects

The percentages of auditory, visual, and fusion responses were computed relative to the total amount of responses to McGurk stimuli. The data have been averaged over the two plosive stimuli, and over the two fricative stimuli.

**Figure 2:**
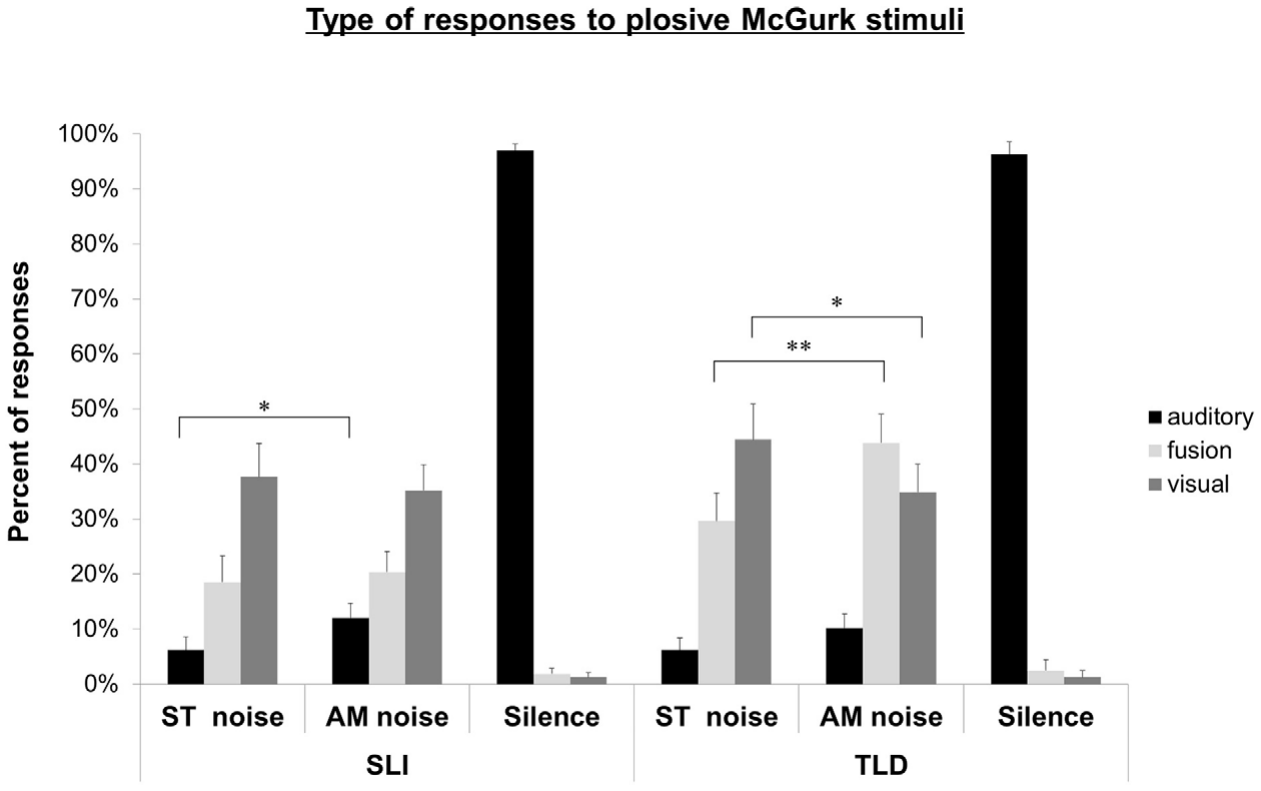
**Experiment 2**. Auditory, fusion, and visual responses to McGurk plosive stimuli for SLI and TLD groups in ST noise, AM noise and quiet conditions. ^*^p < 0.05; ^**^p < 0.01.

***Plosive McGurk stimuli.*** The distribution of responses is shown in Figure 2. In ST noise, TLD children gave 6.2% (SD: 11.5%) auditory responses, 29.6% (SD: 26.3%) of fusions, and 44.4% (SD: 33.3%) of visual responses. Compared to ST noise, their percent of auditory responses (10.2%, SD: 13.3%) did not change; their percent of fusion responses (43.8%, SD: 27.4%) increased, *F*_(1, 26)_ = 8.03, *p* < 0.01; their percent of visual responses (34.9%, SD: 26.5%) significantly decreased, *F*_(1, 26)_ = 4.2, *p* = 0.05.

In ST noise, children with SLI gave 6.2% (SD: 12.4%) of auditory responses, 18.5% (SD: 24.6%) of fusions, and 37.6% (SD: 31.6%) of visual responses. Compared to ST noise, their auditory responses increased in AM noise, [12.0%; SD: 13.9%, *F*_(1, 26)_ = 5.46, *p* < 0.05], but their rate of fusions (20.4%; SD: 19.2%) and visual responses (35.2%, SD: 24.3%) remained unchanged.

Compared to children with TLD, children with SLI had lower fusion responses in AM noise, *F*_(1,52)_ = 13.25, *p* < 0.001.

**Figure 3:**
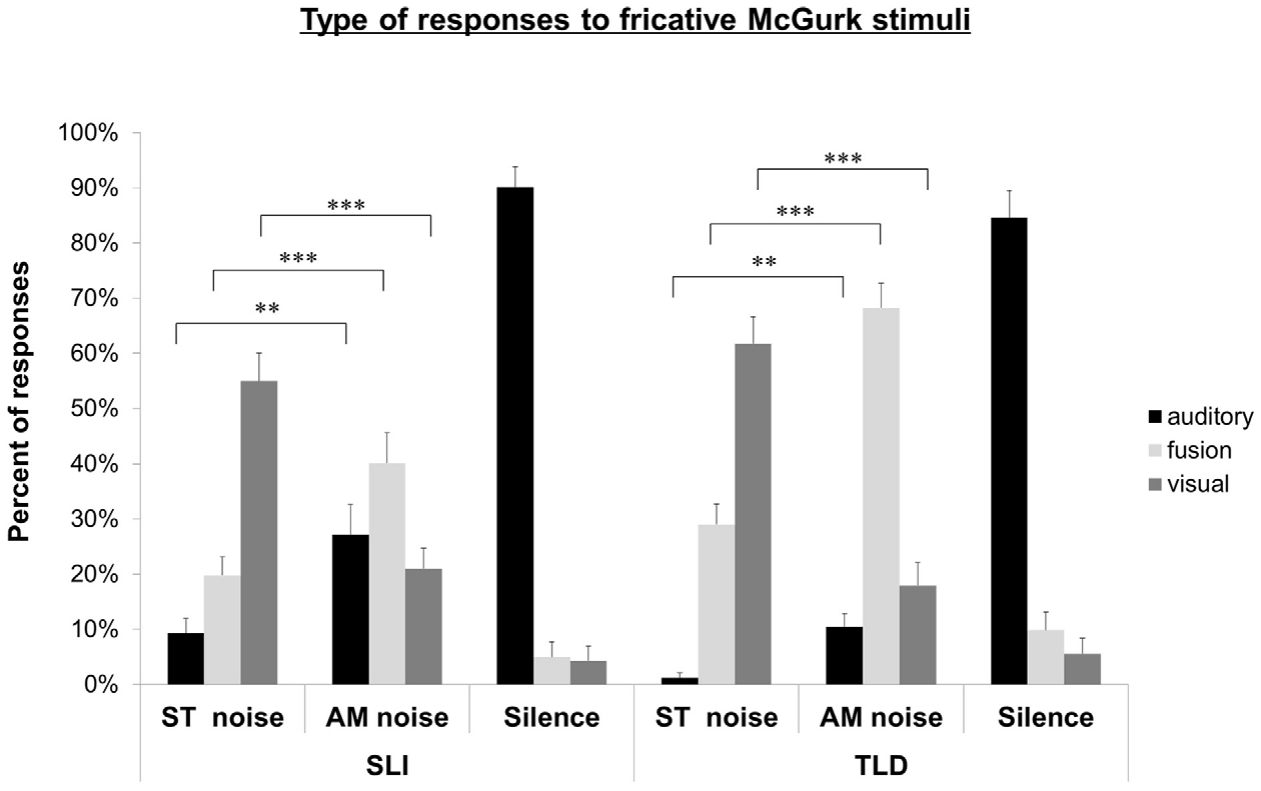
**Experiment 2**. Auditory, fusion, and visual responses to McGurk fricative stimuli for SLI and TLD groups in ST noise, AM noise and quiet conditions. ^**^p < 0.01; ^***^p < 0.001.

***Fricative McGurk stimuli.*** The distribution of responses to fricative McGurk stimuli in the children with SLI group and the children with TLD is shown in Figure 3. In ST noise, TLD children gave 1.2% (SD: 4.4%) of auditory responses, 29.0% (SD: 19.4%) of fusions, and 61.7% (SD: 25.2%) of visual responses. Compared to ST noise, TLD children gave a larger number of auditory responses in AM noise [10.5%, SD: 12.1%, *F*_(1, 26)_ = 11.93, *p* < 0.005], and a larger number of fusion [68.2%, SD: 23.2%, *F*_(1, 26)_ = 132.04; *p* < 0.001]; their rate of visual responses significantly decreased [17.9%, SD: 22.0%, *F*_(1, 26)_ = 125.12; *p* < 0.001].

In ST noise, children with SLI gave 9.3% (SD: 14.1%) of auditory responses, 19.5% (SD: 17.9%) of fusions, and 54.9% of visual responses (SD: 26.5%). Compared to ST noise, they gave significantly more auditory [27.2%; SD: 28.3%; *F*_(1, 26)_ = 9.66; *p* < 0.005], and fusion responses [40.1%; SD: 28.9%; *F*_(1, 26)_ = 16.22; *p* < 0.001]; their rate of visual response significantly decreased [20.9%; SD: 19.25%; *F*_(1, 26)_ = 61.37; *p* < 0.001].

Compared to children with TLD, children with SLI gave more auditory responses in ST and AM noise, *F*_(1, 52)_ = 7.93; *p* < 0.01 and *F*_(1, 52)_ = 7.88, *p* < 0.01 respectively, and less fusions in AM noise, *F*_(1, 52)_ = 15.42, *p* < 0.001. No difference appeared for visual responses.

Finally, in quiet, there was no difference between children with SLI and children with TLD for any kind of response (see Figures 2, 3).

To sum up, the pattern of responses to both plosive and fricative McGurk stimuli was clearly modified by the degree of degradation of auditory information in children with TLD. Compared to ST noise, AM noise decreased the rate of visual responses, and increased auditory and fusion responses. For children with SLI, the pattern was more mixed. AM noise increased the rate of auditory responses for both plosive and fricative, in coherence with their intact auditory masking effect. AM noise increased the rate of fusions, and decreased the rate of visual responses only in the context of fricatives, when the visual information is easily identified. These latter observations are indicative of the audiovisual integration ability of children with SLI.

#### Phonetic feature information transmission (IT)

**Figure 4:**
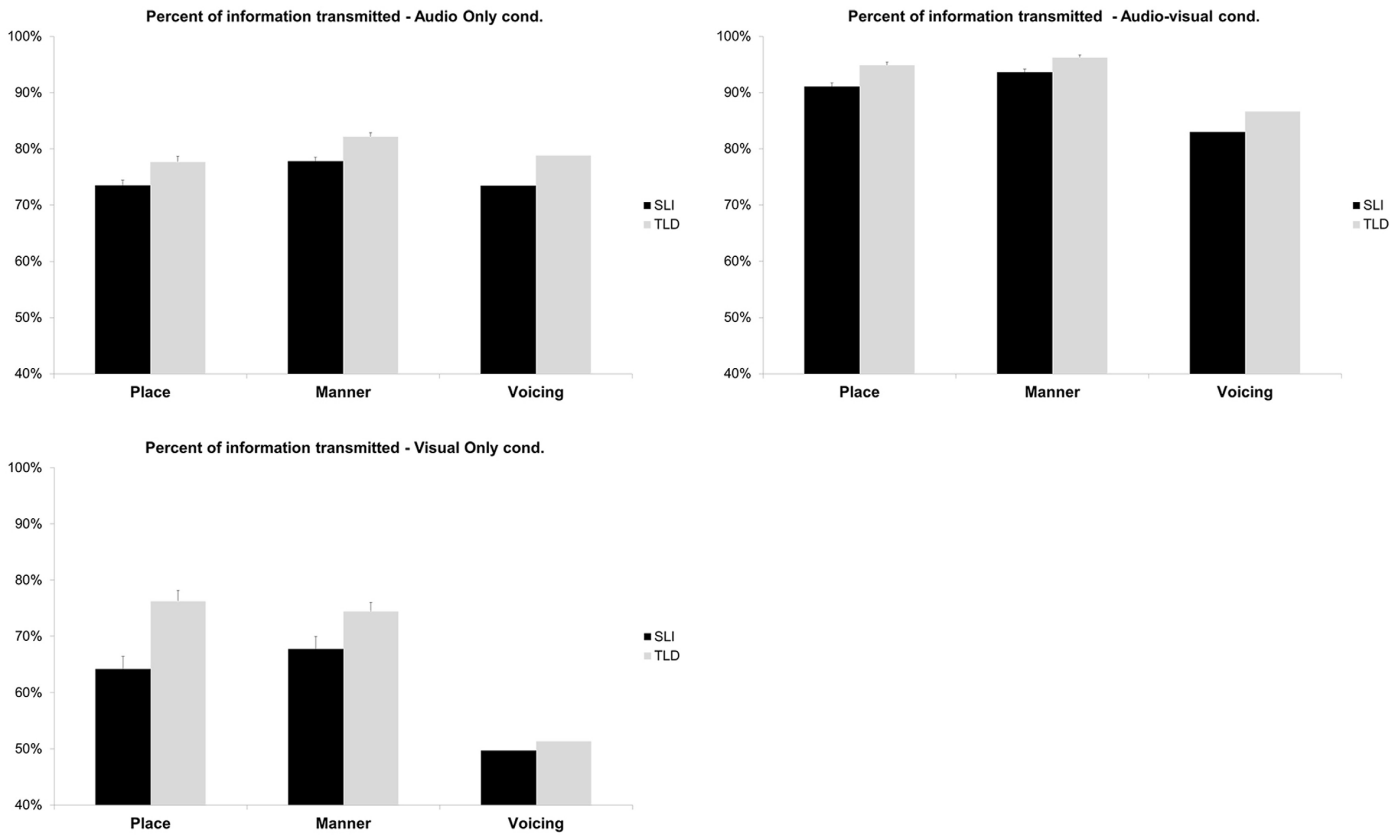
**Percent of information transmitted for place, manner, and voicing, as a function of group (SLI vs. TLD) and modality (auditory, audiovisual, and visual)**.

The reception of place, manner and voicing features was evaluated by information transmission (IT) analyses performed on the basis of the individual confusion matrices. The percent of IT was averaged over quiet, ST noise and AM noise, and displayed in Figure 4.

Because IT was very different in AO or AV than in VO, two separate analyses were run. A repeated measures ANOVA with Feature (place, manner, voicing) and Modality (AO, AV) as within subjects factor, and Group as between subjects factor yielded significant effects of features, *F*_(2, 104)_ = 111.41 *p* < 0.001, of Modality, *F*_(1, 54)_ = 925.33, *p* < 0.001, and of Group, *F*_(1, 52)_ = 35.15, *p* < 0.001. The Modality × Features interaction was significant, *F*_(2, 104)_ = 102.22, *p* < 0.001: IT increases from AO to AV was 17.4% for place, 14.9% for manner, and 8.7% for voicing. No other interaction was significant.

The percent of IT in VO was analyzed with a repeated measures ANOVA with Features (place, manner, voicing) as within subjects factor, and Group as between subjects factor. The analysis yielded a significant effect of Features, *F*_(2, 104)_ = 190.86, *p* < 0.001, and of Group, *F*_(1, 52)_ = 11.58, *p* < 0.001. The Group by Feature interaction was also significant, *F*_(2, 104)_ = 9.42, *p* < 0.001: the difference between groups was large for place of articulation (12.0%), intermediate for manner (6.6%) and almost null for voicing (1.7%).

### Discussion

In Experiment 2, new groups of children with SLI and children with TLD matched as closely as possible for gender, chronological age and non-verbal intelligence were tested with an audio-visual masking release paradigm. The identification of syllables in noise was clearly more difficult in Experiment 2 than in Experiment 1. Two reasons may be invoked. A more extensive protocol consisting of voiced and voiceless syllables was administered, and the voiced syllables were more difficult to identify than the voiceless ones. In addition, children with SLI of Experiment 2 could be more language impaired than those of Experiment 1, as indicated by their lower scores on the Word Repetition test.

Despite these differences, the main results of Experiment 2 remarkably replicated the findings of Experiment 1. Children with SLI showed a speech-in-noise deficit, but a masking release effect comparable in size to that of children with TLD. A clear speechreading deficit appeared in children with SLI compared to TLD children. Children with SLI also had less accurate audio-visual speech perception than TLD children. With no surprise, the standardized visual gains of children with SLI were lower than those of TLD children, coherently with the tendency observed in Experiment 1 (see Table 4). Taken together, the results suggest intact processes at peripheral hearing, but impairment in supra-modal phonemic categorization in children with SLI.

The analysis of percent of information transmitted (IT) is useful to better understand the dynamics of speech perception. A significant increase of percent of IT in AV compared to AO was observed for place of articulation and manner in both groups of children. The complementarity between auditory and visual information is maximal for place of articulation. The fricatives /aӡa/ and /a∫a/, which are highly visible, contribute to the improvement of visual transmission of both manner and place of articulation. Surprisingly, the perception of voicing was improved in AV although voicing was not transmitted by speechreading itself, as indicated by the 50% of IT in the VO modality. It is possible that seeing the movement of the articulators enhances children's attention to the coming sound.

Interestingly, children with SLI showed lower IT percent than children with TLD in the three modalities. This demonstrates that their deficit of IT already found in AO (Ziegler et al., 2005) extends to AV and VO modalities. In VO, the larger difference between the two groups was for place of articulation.

The responses to McGurk stimuli showed an interesting contrast between plosives and fricatives. In the case of plosives A/apa/V/aka/ and A/aba/V/aga/, the visual syllables were poorly identified: 48% by TLD children, and 42% by SLI children. V/aka/ and /aga/ are often confused with V/ata/ and /ada/ (19% in TLD, and 15% in SLI), as well as with V/asa/ and /aza/ (17% in TLD and 19% in SLI). Therefore, the visual information transmitted is reduced and the percent of visual responses is low for both groups. When more auditory information became available, TLD children showed an increase of fusions. SLI children did not but increased their rate of auditory responses.

In the case of fricatives A/afa/V/a∫a/ and A/ava/V/aӡa/, the visual information is easily identified: 84% in TLD and 65% in SLI children. Both groups showed a large amount of visual responses in ST noise (62% for TLD and 55% for SLI children), which significantly decreased in AM noise (18% in TLD and 21% in SLI children). Both groups also showed an increase of auditory and fusion responses in AM, when more auditory was available. The data thus suggest that when children with SLI get access to visual information, they are able to integrate it with auditory information. In other words, the pattern of responses of SLI children is the result of their poorer lipreading skills, but not of a deficit in AV integration. Taken together, the data illustrate the interest of using two types of McGurk stimuli, varying by the degree of availability of the visual information (Berthommier, 2001).

## General discussion

The aim of the present studies was to test to what extend children with SLI make use of visual articulatory cues to improve their speech-in-noise perception. We used a masking release paradigm, with syllables embedded in ST and AM noise, to which we added the visual information of a talking face (Huyse et al., 2012). Syllables were presented to the participants in three modalities: AO, VO, and AV (congruent and incongruent). We also measured the consonant identification in AO, VO, and AV in quiet. We used child-friendly procedures to elicit the responses to the syllables, i.e., to designate a letter corresponding to the consonant they thought the speaker had said, or to immediately repeat the syllable.

Children with SLI and their age-matched control children performed at, or near, ceiling level when asked to identify syllables in AO or in AV congruent when stimuli were presented in quiet. Our results clearly demonstrate an absence of (or only subtle) difficulty for children with SLI in discriminating /aCa/ syllables under optimal listening conditions. These data confirm previous studies testing AO (Ziegler et al., 2005) or AV speech perception (Norrix et al., 2007; Meronen et al., 2013). Our speech stimuli were produced naturally. Natural speech is rich in redundant acoustic cues and may be easier for children with SLI to perceive than synthetic stimuli (Evans et al., 2002). The good results obtained by children with SLI under optimal conditions also validate our response procedures, which are resistant to decay from phonological short-term memory. Thus, we might be confident that the simple task demands, combined with natural speech, allow us to accurately assess the identification of /aCa/ tokens by SLI and age-control children.

We predicted that children with SLI would experience a speech-in-noise deficit but an intact masking-release effect in the auditory modality (Ziegler et al., 2005). Both expectations were confirmed in Experiments 1 and 2. Contrasting with their good performance under optimal listening conditions, children with SLI show a marked deficit in noisy conditions, confirming their difficulties in separating speech from noise (Sperling et al., 2005; Hornickel et al., 2009; Ziegler et al., 2009). Children with SLI showed a masking release effect of the same size (around 30%) than that of control children. An intact masking release effect is usually taken as a signature of appropriate use of the short temporal minima in the fluctuating background to perceive speech cues, suggesting that the “sensory and cognitive processes known to be involved in masking release, such as auditory grouping based on stimulus spectral and fine-structure cues, perceptual restoration, and informational masking, are functional in children with SLI” (Ziegler et al., 2005, p. 14113). Our data thus support the claim that an intact masking release despite a deficit in speech-in-noise constitutes a robust effect in children with SLI.

If developmental SLI reflects a dysfunction in phonemic categorization as opposed to a purely auditory disorder, we could expect to observe a speechreading deficit. The results of Experiments 1 and 2 clearly showed that children with SLI were less accurate than TLD children in identifying the consonants belonging to six different visemes. Therefore, when speech-in-noise deficit is due to central processing dysfunctioning (rather than to peripherally based auditory problem as in cochlear implantees, see Huyse et al., 2012), the deficit is amodal, and children are less accurate in identifying visual articulatory cues (De Gelder and Vroomen, 1998; Ramirez and Mann, 2005; Norrix et al., 2007; Leybaert and Colin, 2008; Meronen et al., 2013).

Not surprisingly, children with SLI were less influenced by the visual speech cues than TLD children. Clear differences appeared in how participants effectively used visual cues to recover place of articulation when /aCa/ syllables were masked by noise. Children with SLI had lower visual gains both in ST and AM noise (significantly in Experiment 2 and quantitatively in Experiment 1). Again, this result dismisses the idea that the speech perception deficit of children with SLI has a purely auditory basis. Should that be the case, the deficit in the auditory processing domain could be partially circumvented by reliance on visual speech.

The speechreading deficit of SLI children also impacts their response pattern to McGurk stimuli. As expected, TLD children gave mainly visual responses in ST noise, and significantly more auditory and fusions responses in AM noise. In other words, TLD children exhibited a release from masking of the McGurk fusions (Huyse et al., 2012). Children with SLI gave significantly less visual responses than the controls in ST noise (Experiment 1), and less fusions in AM noise (Experiments 1 and 2), confirming previous data (Norrix et al., 2007; Leybaert and Colin, 2008; Meronen et al., 2013).

How to explain the pattern of responses of SLI children to McGurk stimuli ? Do the responses of SLI children result from their lower speechreading skills, or, alternatively, are they the consequence of an atypical integration process itself? On one hand, when visual information is clearly available (as in the fricatives of Experiment 2), children with SLI seem able to integrate auditory and visual information adequately, even if they showed less influence of visual speech. This result is compatible with the “deficit in speechreading skills” hypothesis. On the other hand, it may be that the visual articulatory gestures are processed more independently of the auditory information for children with SLI than for children with TLD. Green (1998) suggested that young children might weight auditory dimensions differently than older children, and alternative weighting might result in reduced interaction with the visual information. Thus, children with SLI may differ from their peers with TLD in terms of how they weight the visual dimensions of the articulated speech segments. We are presently running a new experiment to test more directly these two hypotheses.

The data obtained by children with SLI contrast with those obtained by children with cochlear implant assessed with a similar paradigm (Huyse et al., 2012). In deaf children fitted with a CI, a peripherally based disorder underlies deficits in auditory speech processing in noise; this deficit could be partially circumvented by the introduction of visual articulatory cues. By contrast, a central, amodal deficit in phonemic categorization prevents children with SLI from effectively utilizing these visual articulatory cues. In future, it would be interesting to investigate whether this difference helps identify CI children with SLI.

There are several limitations to the present studies. Children with SLI are a heterogeneous group. It would be interesting to examine whether their speechreading ability and use of visual cues to improve audiovisual speech perception is also variable. Do they differ in linguistic processing of visible articulatory gestures, or do they differ in attentional processes ? Is there a relation between impairment in visible speech processing and potential temporal processing deficits in SLI (see Ten Oever et al., 2013, for a discussion about how AV timing information on articulatory cues aids in syllable identification)?

In addition, the deficit in speech-in-noise perception, poor perception of visual speech, difficulties in fusing auditory and visual stimuli in classic McGurk stimuli could be related to cortical and sub-cortical responses in future studies. According to Hornickel et al. (2009), abnormal encoding of the place of articulation feature of stop consonants should appear in the auditory brainstem in children with SLI. Such a deficit in the encoding of formant information would lead to representations less resistant to noise, and, possibly, to an under-development of the processing of place of articulation in visual speech, and of integration of auditory and visual speech. In addition, audio-visual integration also has a corresponding cortical response (Colin et al., 2002), which could be absent or reduced in children with SLI.

We can only speculate as to whether or not language training may modify the ability of children with SLI to process the visual speech cues. A long-term study on how speech and language remediation training can help children with SLI more effectively utilize visual articulatory cues in identifying impoverished speech elements may help address this issue better. It would also be interesting to investigate whether their reduced ability to combine auditory and visual information is speech specific, or also occur for other types of integration auditory and visual non-speech, or audio-tactile information. This issue is at the agenda for future research. The outcomes of these types of research will help to better understand the causes of reduced audio-visual speech integration in children with SLI, and to design more adapted rehabilitation programs.

### Conflict of interest statement

The authors declare that the research was conducted in the absence of any commercial or financial relationships that could be construed as a potential conflict of interest.
